# Erythrocyte and Platelet Indices at Admission and Discharge Stratify Long-Term Cardiovascular Risk After Invasive Treatment of Myocardial Infarction

**DOI:** 10.3390/jcm15124455

**Published:** 2026-06-09

**Authors:** Christoph Strohhofer, Faisal Aziz, Andreas Kainz, Andrea Berghold, Nicolas Verheyen, Heiko Bugger, Stefan Hatzl, Raffaela Planka, Friederike von Lewinski, Johannes Gollmer, Klemens Ablasser, Ivan Vosko, Michael Sacherer, Gabor G. Toth, Ewald Kolesnik, Andreas Zirlik, Harald Sourij, Dirk von Lewinski

**Affiliations:** 1 Division of Cardiology, Department of Internal Medicine, Medical University of Graz, 8010 Graz, Austria; 2 Division of Endocrinology and Diabetology, Department of Internal Medicine, Medical University of Graz, 8010 Graz, Austria; 3 Institute for Medical Informatics, Statistics and Documentation, Medical University of Graz, 8010 Graz, Austria; 4 Division of Hematology, Department of Internal Medicine, Medical University of Graz, 8010 Graz, Austria; 5 Department of Microbiology, Icahn School of Medicine at Mount Sinai, New York, NY 10029, USA; 6 Interdisciplinary Metabolic Medicine Trials Unit, Medical University of Graz, 8010 Graz, Austria

**Keywords:** myocardial infarction, acute coronary syndrome, hemoglobin, red blood cell indices, platelets, prognostic biomarkers

## Abstract

**Background/Objectives**: Cardiovascular risk remains substantial after myocardial infarction (MI) despite established clinical risk markers. Erythrocyte and platelet indices are routinely available, but their long-term prognostic relevance remains insufficiently studied. **Methods**: This retrospective cohort study was based on the Styrian Registry on Genuine Myocardial Infarction (STRONG-MI) and included patients with MI undergoing invasive coronary angiography in Styria, Austria, between January 2007 and March 2016. Multivariable Cox regression models were used to assess the associations of admission and discharge erythrocyte and platelet indices with 3-point major adverse cardiovascular events (MACE) during follow-up extending to 175 months. **Results**: Among 10,920 patients, admission hemoglobin showed a U-shaped association with MACE. Median hemoglobin decreased from admission to discharge (14.2 g/dL vs. 13.1 g/dL) and the lowest discharge tertile showed the highest association with MACE compared with the middle tertile (AHR 1.27, 95% CI 1.18–1.38). Lower mean corpuscular hemoglobin concentration (MCHC) was independently linked to adverse outcomes at both admission and discharge (AHR 1.17, 95% CI 1.08–1.27, and 1.14, 95% CI 1.05–1.23, respectively). Higher platelet count and mean platelet volume (MPV) were also associated with increased risk, particularly at discharge (AHR 1.15, 95% CI 1.06–1.24, and 1.19, 95% CI 1.10–1.29, respectively). **Conclusions**: Routine erythrocyte and platelet indices were independently associated with long-term cardiovascular outcomes after invasively treated MI and reflect residual biological risk after discharge.

## 1. Introduction

Myocardial infarction (MI) remains a major cause of morbidity and mortality despite advances in revascularization and secondary prevention. Risk stratification after MI relies largely on established demographic and cardiometabolic factors, yet widely available biomarkers may provide additional prognostic information [1,2,3]. Routine erythrocyte and platelet indices are universally available and biologically linked to oxygen transport and thrombosis, but their long-term prognostic value over many years after MI remains incompletely defined [4,5].

Between 10.5% and 46.4% of patients presenting with acute coronary syndrome (ACS) are anemic and have an increased risk of reinfarction and all-cause mortality as early as 30 days after ACS, with excess risk persisting for at least one year in large-scale meta-analyses [6,7,8,9]. Patients with anemia are also less likely to receive evidence-based therapies [10,11]. Importantly, excess risk has persisted even among optimally treated ACS patients undergoing early invasive strategies with high PCI success rates and contemporary lipid-lowering therapy [12,13,14,15]. Conversely, high hemoglobin levels have also been linked to increased mortality in population-based cohorts, suggesting a U-shaped relationship between hemoglobin concentration and cardiovascular risk [16].

Beyond hemoglobin, the prognostic significance of other routine erythrocyte indices, including mean corpuscular volume (MCV), mean corpuscular hemoglobin (MCH), and mean corpuscular hemoglobin concentration (MCHC), is less well established. These indices reflect erythrocyte morphology and hemoglobinization and may provide information beyond hemoglobin alone. Prior studies have reported associations between erythrocyte indices and adverse outcomes after MI, but findings have been inconsistent and were often derived from selected populations or shorter follow-up [17,18].

Platelets represent another central axis in the pathophysiology of MI. Routine platelet indices such as platelet count and mean platelet volume (MPV) may reflect thrombotic burden and platelet reactivity, but their prognostic relevance in MI is less consistently defined than that of hemoglobin. Moreover, most previous studies have focused on admission values and short-term outcomes, whereas data on discharge platelet indices and long-term cardiovascular risk remain limited [19,20,21]. Large-scale multicenter data are therefore needed to evaluate the long-term prognostic relevance of these routinely available indices.

## 2. Materials and Methods

### 2.1. Study Design and Population

The Styrian Registry on Genuine Myocardial Infarction (STRONG-MI) systematically includes all patients with myocardial infarctions who were referred for coronary angiography in Styria, Austria, between January 2007 and March 2016. To ensure comprehensive regional coverage, data from all three interventional cardiology centers in Styria were included: University Hospital Graz; LKH Graz II, West location; and LKH Hochsteiermark, Location Bruck an der Mur. These centers represent the only facilities performing acute invasive coronary procedures in a region of approximately 1,200,000 inhabitants. The classification of MI subtypes for inclusion into the registry was based on clinical presentation, ECG findings, and biomarker assessment. ST-elevation myocardial infarction (STEMI) was defined by acute chest pain (or equivalent symptoms) and persistent ST-segment elevation (or its equivalents) on ECG, fulfilling the universal definition of MI at that time [22]. Patients with acute chest pain but without persistent ST-segment elevation were further categorized based on high-sensitivity cardiac troponin (hs-cTn) levels. NSTEMI was diagnosed if hs-cTn levels showed a significant rise and/or fall above the 99th percentile, indicating myocardial necrosis. All patients included in the STRONG-MI registry were analyzed, irrespective of whether both admission and discharge hemogram values were available. Missing discharge hemogram values were handled within the multiple imputation framework. The study was conducted under the approval by the Ethics Committee of the Medical University of Graz (EK No: 28-433 ex15/16, 1200/2016) for the parent registry, and data were collected in accordance with ethical and regulatory guidelines.

### 2.2. Follow-Up and Study Endpoints

Time-to-event follow-up began at the date of coronary angiography during the index hospitalization and continued until the first occurrence of the outcome of interest or administrative censoring at the last available date of outcome follow-up. Follow-up events were identified through ICD-coded diagnoses from the regional hospital information system (HIS) openMEDOCS. Mortality data were obtained from Statistics Austria, the official national source for mortality statistics in Austria. Cause of death was based on official death certificates completed by the responsible examining physicians and was used to classify cardiovascular and non-cardiovascular mortality according to ICD-10 codes. The 3-point MACE endpoint was defined as cardiovascular mortality (ICD-10 codes I00–I78), recurrent MI (ICD-10 codes I21.0–I21.4, I21.9), and stroke (ICD-10 codes I61, I63, I64). The data were extracted by the Institute for Medical Informatics, Statistics and Documentation of the Medical University of Graz and provided in accordance with data protection laws on internal servers. To improve endpoint validity and avoid double counting of recurrent MI across hospitals due to interhospital transfers or rehabilitation stays, all recurrent MI diagnoses not confirmed by inclusion in the STRONG-MI registry were individually reviewed and adjudicated by the first author, a clinician in internal medicine and cardiology. This review was based on ICD-coded diagnoses from openMEDOCS and available clinical information, including timing of diagnosis, ECG findings and biomarker assessment.

### 2.3. Laboratory Variables

Laboratory data were collected as part of routine clinical care during the index hospitalization. Therefore, admission values were generally obtained during the acute presentation, whereas discharge values were drawn according to clinical need, often shortly before discharge but without a standardized protocol. The first available measurement was defined as the admission value, and the last available measurement as the discharge value. The term discharge value was used operationally and refers to the last available in-hospital hemogram measurement, including patients without a formal discharge record. The present analysis focused on erythrocyte and platelet indices derived from the routine blood count, including hemoglobin, erythrocyte count, hematocrit, MCH, MCHC, MCV, platelet count, and MPV. Because identical reference ranges were used across hospitals within the Styrian Hospital Association, data could be merged across centers. Parameters primarily reflecting systemic inflammation, such as white blood cell count, were not included because they represent a distinct biological domain from erythrocyte and platelet indices.

### 2.4. Statistical Analysis

Statistical analyses were performed using R version 4.4.2. Data are reported as frequency and percentage (%) for categorical variables and mean ± standard deviation (SD) or median and interquartile range (IQR) for continuous variables. Comparisons according to MACE status were performed using the chi-square test for categorical variables and the Wilcoxon rank-sum test for continuous variables. The incidence probabilities were estimated using Kaplan–Meier curves, and log-rank tests were applied to compare probability curves by hemogram tertiles. Incidence rates and cumulative incidence of composite and individual MACE with respect to hemogram tertiles were calculated at prespecified time points. Cox proportional-hazards regression models were used to ascertain the association of tertile-based hematological variables with the risk of composite and individual MACE. The proportional hazards assumption was assessed using Schoenfeld residuals. Fine-Gray competing-risk models were used as sensitivity analyses for recurrent MI and stroke, treating all-cause death before the respective non-fatal event as the competing event. Univariable models were used to estimate unadjusted associations of each hematological variable with MACE. In multivariable Cox regression, the association of hematological variables with MACE was adjusted for age, sex, BMI, hyperlipidemia, MI type, previous MI, previous stroke, diabetes, multiple- versus single-vessel PCI, number of baseline comorbidities, and eGFR. To explore the incremental discriminatory value of hematological indices, additional time-dependent receiver operating characteristic (ROC) analyses were performed. The clinical base model included age, sex, BMI, hyperlipidemia, MI type, previous MI, previous stroke, diabetes, multiple- versus single-vessel PCI, number of baseline comorbidities, and eGFR. The number of baseline comorbidities was defined as an unweighted count of ICD-10-coded hypertension, hyperlipidemia, diabetes mellitus, chronic obstructive pulmonary disease, atrial fibrillation or flutter, and asthma documented in openMEDOCS. Extended models additionally included one selected hematological marker at a time. Exploratory model discrimination was assessed using time-dependent area under the curve (AUC) at 60 and 120 months. Given the exploratory nature of these analyses, ROC/AUC results were used to describe potential incremental discrimination and were not intended to develop or validate a clinical prediction model. Missing data were managed using multivariate imputation with the multivariate imputation by chained equations (MICE) package in R. Imputations were performed separately for admission and discharge hemogram measurements. The imputation models included demographic and clinical variables, hemogram variables (missing and non-missing), and outcome variables. The Nelson-Aalen cumulative hazard estimator was used as an auxiliary predictor in the imputation models to account for the time-to-event structure of outcomes. Variables were imputed using predictive mean matching (PMM). For each imputation model, 40 imputed datasets were generated using 20 iterations. Convergence was assessed using trace plots, and plausibility of imputations was assessed using density plots. Subsequent analyses were performed across the imputed datasets according to Rubin’s rules. To assess robustness, complete-case sensitivity analyses were reported for the multivariable Cox models. Because the evaluated hematological indices are biologically interrelated, the primary interpretation was based on coherent patterns across related parameters rather than on isolated *p*-values. Given the number of markers, Benjamini–Hochberg false-discovery-rate adjustment was additionally applied to the main tertile-based MACE analyses as a sensitivity assessment, and both nominal and FDR-adjusted *p*-values are reported.

## 3. Results

The study included 10,920 patients with MI (Table 1). NSTEMI was more frequent than STEMI (62% vs. 38%). Most patients were male (65%; n = 7116), the mean age was 66.6 ± 12.7 years, and median body mass index (BMI) was 27.0 kg/m^2^ (IQR 24.4–30.0). Hypertension (72%; n = 7889) and hyperlipidemia (44%; n = 4825) were the most prevalent cardiovascular risk factors, followed by diabetes mellitus in 25% (n = 2712). Chronic obstructive pulmonary disease was present in 8.7% (n = 945), and asthma in 1.3% (n = 140). Median estimated glomerular filtration rate (CKD-EPIBSA) was 73.8 mL/min/1.73 m^2^ (IQR 54.2–89.7). The median length of hospital stay was 8 days (IQR 5–13).

At admission, median hemoglobin was 14.2 g/dL (IQR 13.0–15.3), median erythrocyte count was 4.6 million/µL (IQR 4.3–5.0), and median hematocrit was 41.5% (IQR 38.3–44.2). Median MCV was 89.0 fL (86.2–92.1), MCH was 30.5 pg/cell (IQR 29.3–31.7) and MCHC was 34.0 g/dL (IQR 33.3–35.0). Median MPV at admission was 10.0 fL (IQR 9.0–10.8). At discharge, median hemoglobin decreased to 13.1 g/dL (IQR 11.7–14.3) and erythrocyte count to 4.32 million/µL (IQR 3.9–4.7), whereas platelet count remained largely stable, increasing slightly from 228.0 × 10^9^/L (IQR 190.0, 273.0) to 234.0 × 10^9^/L (IQR 190.0, 296.0). Median MPV was 10.1 fL (IQR 9.1–11.0) at discharge. Tertile cutoffs for all hematological indices are shown in Table S1. To facilitate interpretation of the main findings, hemoglobin tertiles were approximately <13.0, 13.0–15.3, and >15.3 g/dL at admission and <11.7, 11.7–14.3, and >14.3 g/dL at discharge. Corresponding MPV tertiles were <9.0, 9.0–10.8, and >10.8 fL at admission and <9.1, 9.1–11.0, and >11.0 fL at discharge.

The cumulative incidence of 3-point MACE was 12.3% at 1 year and 24% at 5 years. At 1 year, cumulative incidences were 4.3% for recurrent MI and 1.4% for stroke, while all-cause and cardiovascular mortality were 10.5% and 7.3%, respectively. During the overall follow-up of 103 months (IQR 69–138), extending to 175 months, 31.5% of patients experienced a 3-point MACE (Table S2). All-cause mortality occurred in 36% of the cohort and cardiovascular mortality in 18%, whereas non-fatal recurrent MI and non-fatal stroke were observed in 11.5% and 7%, respectively.

**Figure 1:**
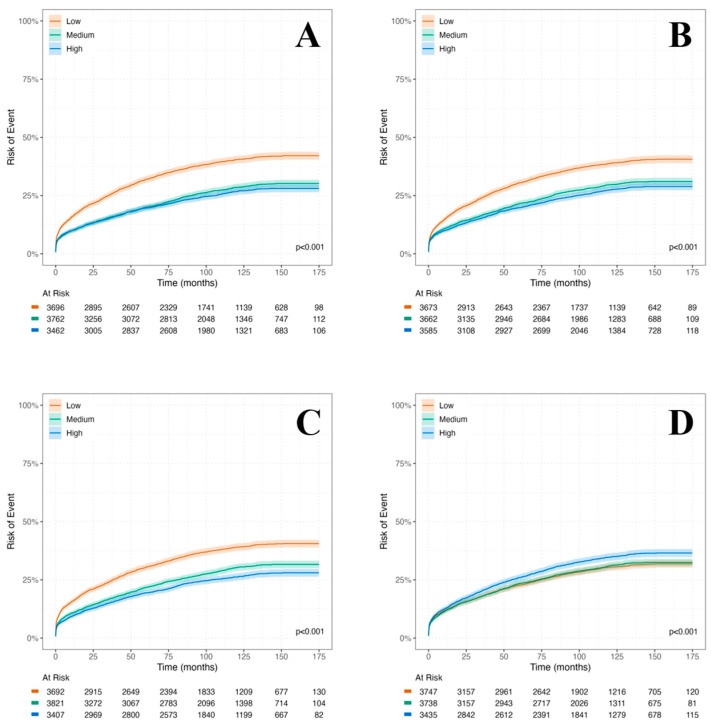
Kaplan–Meier estimates of cumulative event probability for 3-point MACE by admission tertiles. (**A**) hemoglobin, (**B**) erythrocyte count, (**C**) MCHC, and (**D**) MPV at admission are shown in three groups (low, medium, high). Abbreviations: MACE, major adverse cardiovascular events; MCH, mean corpuscular hemoglobin; MCHC, mean corpuscular hemoglobin concentration; MCV, mean corpuscular volume; MPV, mean platelet volume.

Kaplan–Meier analyses showed higher cumulative event rates in the lowest tertiles of several erythrocyte indices, most consistently for hemoglobin at admission (Figure 1) and discharge (Figure S1) and for discharge erythrocyte count and hematocrit. In contrast, platelet-related risk was highest in the upper tertiles, particularly for discharge MPV. As shown in Figure S2, the excess risk linked to low hemoglobin was largely driven by cardiovascular death, whereas the effects related to tertiles of platelet indices were more evenly distributed across individual MACE components.

In multivariable Cox regression analyses adjusted for age, sex, BMI, hyperlipidemia, MI type, prior MI, prior stroke, diabetes, multiple- versus single-vessel PCI, number of baseline comorbidities, and eGFR, admission and discharge hematological indices showed distinct associations with 3-point MACE (Table 2). Assessment based on Schoenfeld residuals showed no relevant violation of the proportional hazard assumption for the main Cox models.

**Figure 2:**
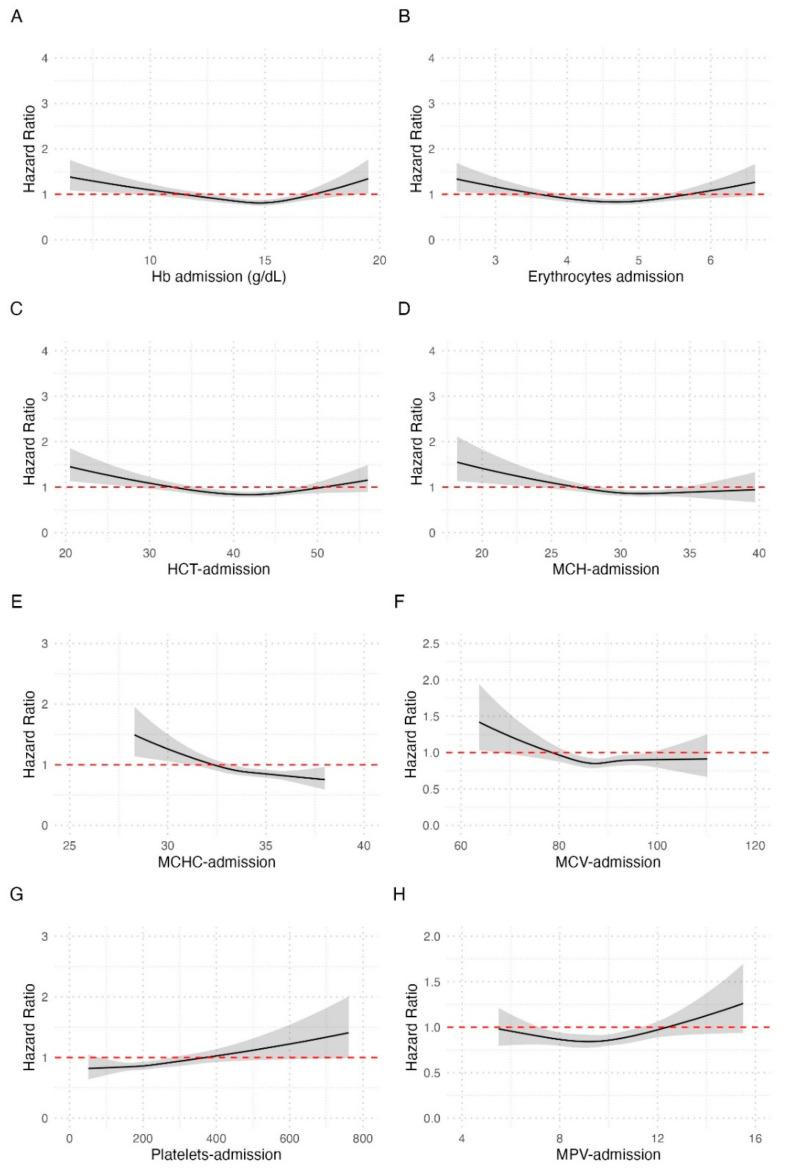
Adjusted hazard ratios of 3-point MACE for hemogram values at admission. (**A**) hemoglobin, (**B**) erythrocyte count, (**C**) hematocrit, (**D**) MCH, (**E**) MCHC, (**F**) MCV, (**G**) platelet count, and (**H**) MPV. Abbreviations: Hb, hemoglobin; HR, hazard ratio; MACE, major adverse cardiovascular events; MCH, mean corpuscular hemoglobin; MCHC, mean corpuscular hemoglobin concentration; MCV, mean corpuscular volume; MPV, mean platelet volume.

At admission, hemoglobin showed a clear U-shaped relationship with 3-point MACE (Figure 2). Compared with the middle tertile, both the lowest and highest tertiles carried increased risk (AHR 1.19, 95% CI 1.09–1.29 and AHR 1.11, 95% CI 1.02–1.22, respectively). In addition, lower hematocrit, erythrocyte count, MCH, and MCHC were associated with higher risk, whereas MCV showed no relationship with 3-point MACE.

At discharge, erythrocyte-related associations were stronger and more consistent (Figure S3). Lower hemoglobin, erythrocyte count, hematocrit, and MCHC all identified patients at higher risk of 3-point MACE. The strongest risks were observed for low hemoglobin (AHR 1.27, 95% CI 1.18–1.38) and low erythrocyte count (AHR 1.24, 95% CI 1.14–1.34). MCV again showed no association.

In contrast, platelet-related associations were concentrated in the upper tertiles. Higher platelet count was associated with increased risk at both admission and discharge when analyzed by tertiles (admission high tertile: AHR 1.11, 95% CI 1.02–1.21; discharge high tertile: AHR 1.15, 95% CI 1.06–1.24). Similarly, MPV was linked to an increased risk in the highest tertile at admission (AHR 1.09, 95% CI 1.01–1.18) and at discharge (AHR 1.19, 95% CI 1.10–1.29).

Missingness was low across covariates and hematological variables (Table S3). Complete-case sensitivity analyses yielded consistent results with the imputed main analyses and are presented in Table S4.

In Fine-Gray competing-risk sensitivity analyses for recurrent MI and stroke, with all-cause death treated as the competing event, the endpoint-specific associations were broadly consistent with the main analyses and did not materially alter the interpretation of the findings (Figure S4). In exploratory time-dependent ROC analyses, the clinical base model discriminated 3-point MACE at 60 and 120 months with AUC values of 0.708 (95% CI 0.697–0.721) and 0.707 (95% CI 0.695–0.719), respectively. Addition of individual hematological markers resulted in similar AUC values (Figure S5) and did not significantly improve model discrimination compared with the clinical base model.

## 4. Discussion

In this large multicenter real-world cohort of 10,920 patients with invasively treated myocardial infarction and follow-up extending to 175 months, routine erythrocyte and platelet indices at admission and discharge were associated with distinct patterns of long-term cardiovascular risk. First, admission hemoglobin showed a U-shaped relationship with 3-point MACE, with excess risk at both low and high values. Second, discharge erythrocyte indices, particularly hemoglobin, erythrocyte count, hematocrit, and MCHC, showed stronger associations with adverse outcomes than admission values. Third, excess risk linked to low hemoglobin was driven predominantly by cardiovascular death rather than by recurrent MI or stroke. Fourth, platelet-related risk followed the opposite direction, with higher platelet count and MPV indicating increased risk. Our findings extend prior evidence linking anemia to adverse outcomes after acute coronary syndromes. Prior studies and meta-analyses have shown that low hemoglobin is associated with worse short- to mid-term outcomes after ACS [6,7,8,9,10,11,12,13,14,15]. However, most previous studies focused either on hemoglobin alone, on admission values only, or on substantially shorter follow-up. The present study extends previous work by evaluating routinely available erythrocyte and platelet indices in a large, region-wide MI cohort with long-term follow-up.

The U-shaped relationship between admission hemoglobin and long-term MACE is biologically plausible. Low hemoglobin may reflect reduced oxygen-carrying capacity, bleeding susceptibility, frailty, or malignant disease. Conversely, high hemoglobin may identify patients with smoking-related erythrocytosis, chronic hypoxemia, or other conditions linked to increased blood viscosity and vascular risk [23]. Admission values may capture chronic baseline status better, whereas discharge values may integrate the cumulative impact of infarct severity, procedural burden, bleeding, hemodilution, and repeated blood sampling during hospitalization, thereby potentially reflecting hospital-acquired anemia. Importantly, hemoglobin and erythrocyte indices may also be influenced by several additional underlying conditions, including iron deficiency, chronic inflammation, malignancy, liver disease, vitamin B12 or folate deficiency, and hematological disorders. Therefore, the observed associations should primarily be interpreted as markers of residual biological vulnerability rather than as direct evidence of causal effects of individual blood count parameters. Although 3-point MACE was chosen as the primary endpoint, the excess risk associated with low hemoglobin was driven predominantly by cardiovascular death. This is clinically important, because it suggests that low erythrocyte reserve after MI may be less a marker of isolated recurrent ischemic events than of broader cardiovascular fragility. The associations of MCHC may be of particular interest, as MCHC reflects erythrocyte hemoglobinization rather than cell size alone.

Platelet-related findings followed a different but equally plausible pattern. Higher platelet count and MPV at both admission and discharge were linked to worse outcomes. This is consistent with the central role of platelet activation in recurrent atherothrombotic events. The stronger association of MPV at discharge may indicate persistent platelet activation beyond the acute phase.

Several limitations must be acknowledged. First, the retrospective design precludes causal inference. Second, some clinically relevant variables, particularly smoking status and medication data, could not be extracted by the HIS in a standardized manner. Smoking may partly explain the increased risk observed at higher hemoglobin levels, for example through smoking-related erythrocytosis or chronic hypoxemia. Medication use, including antiplatelet therapy, anticoagulation, statins, beta-blockers, renin-angiotensin-system inhibitors, and P2Y12 inhibitor selection, may also have influenced long-term outcomes. However, because all patients underwent invasive management at dedicated PCI centers during an era of established guideline-based secondary prevention, most patients were expected to have received care broadly consistent with contemporary treatment. Generalizability to current practice may still be affected by evolving stent technology, antiplatelet strategies, and lipid-lowering therapy. Even if baseline smoking data were available for the entire cohort, it would still likely incompletely reflect long-term exposure after MI, as the index event often triggers smoking cessation. Nevertheless, residual confounding cannot be excluded. Markers that may help to further characterize the etiology of altered erythrocyte indices, such as iron studies or vitamin levels, were also not routinely available in sufficient completeness for robust analysis. Although repeated laboratory measurements were available during hospitalization, the present analysis focused on admission and discharge values rather than full in-hospital trajectories. This approach improves interpretability and clinical applicability, but it may not fully capture dynamic changes over time. Length of hospital stay was added to characterize the clinical interval between admission and discharge measurements. Nevertheless, variability in hospitalization duration and non-standardized timing of the last laboratory assessment may have influenced discharge-based analyses. Discharge hemogram parameters should therefore be interpreted as markers of later in-hospital clinical status associated with subsequent outcomes. Although several hematological indices were independently associated with long-term MACE, their addition to the clinical base model did not substantially improve time-dependent AUC. Therefore, the present study should not be interpreted as a formal prediction-model study.

Overall, this study shows that routine erythrocyte and platelet indices are associated with complementary patterns of long-term cardiovascular risk after invasively treated MI. Notably, discharge erythrocyte indices yielded a more consistent risk signal than admission values, whereas platelet-related risk was consistently concentrated in the upper range. Our findings do not imply that correction of an abnormal laboratory value alone will improve prognosis [24,25]. Rather, these parameters appear to function as accessible markers of underlying biological vulnerability after MI. Their universal availability and negligible cost make them attractive candidate markers for future risk models and for characterizing patients who may merit closer follow-up after hospital discharge.

## Figures and Tables

**Table 1:** Baseline characteristics.

Characteristics	Overall N = 10,920 ^1^	No MACE N = 7480 ^1^	MACE N = 3440 ^1^	*p*-Value ^2^
Age (years)	66.6 ± 12.7	64.3 ± 12.5	71.5 ± 11.6	<0.001
Sex				<0.001
Female	3804 (34.8%)	2492 (33.3%)	1312 (38.1%)	
Male	7116 (65.2%)	4988 (66.7%)	2128 (61.9%)	
Height (cm)	170.0 (164.0, 176.0)	171.0 (165.0, 178.0)	170.0 (163.0, 175.0)	<0.001
Weight (kg)	79.0 (70.0, 90.0)	80.0 (70.0, 90.0)	77.0 (67.0, 87.0)	<0.001
BMI (kg/m^2^)	27.0 (24.4, 30.0)	27.1 (24.5, 30.1)	26.7 (24.0, 29.7)	<0.001
Hypertension	7889 (72.2%)	5237 (70.0%)	2652 (77.1%)	<0.001
Hyperlipidemia	4825 (44.2%)	3240 (43.3%)	1585 (46.1%)	0.007
Diabetes	2712 (24.8%)	1601 (21.4%)	1111 (32.3%)	<0.001
COPD	945 (8.7%)	536 (7.2%)	409 (11.9%)	<0.001
Atrial fibrillation	1437 (13.2%)	747 (10.0%)	690 (20.1%)	<0.001
Asthma	140 (1.3%)	94 (1.3%)	46 (1.3%)	0.728
GFR (CKD-EPI)	73.8 (54.2, 89.7)	76.7 (59.3, 92.3)	58.8 (40.9, 79.1)	<0.001
Type of MI				<0.001
STEMI	4107 (37.6%)	2900 (38.8%)	1207 (35.1%)	
NSTEMI	6808 (62.3%)	4575 (61.2%)	2233 (64.9%)	

^1^ Mean ± SD; Median (Q1, Q3); n (%), ^2^ Wilcoxon rank sum test; Pearson’s Chi-squared test; Fisher’s exact test. Abbreviations: MACE, major adverse cardiovascular event.

**Table 2:** Multivariable Cox regression analysis of MACE with hematological marker tertiles at admission and discharge.

Characteristic	HR	95% CI	*p*-Value	FDR-Adjusted *p*-Value
Hemoglobin tertiles (admission)				
High	1.11	1.02, 1.22	0.023	0.061
Low	1.19	1.09, 1.29	<0.001	<0.001
Hemoglobin tertiles (discharge)				
High	0.91	0.83, 1.00	0.055	0.126
Low	1.27	1.18, 1.38	<0.001	<0.001
Erythrocytes tertiles (admission)				
High	1.08	0.98, 1.18	0.106	0.170
Low	1.12	1.03, 1.22	0.006	0.032
Erythrocytes tertiles (discharge)				
High	0.99	0.90, 1.08	0.755	0.755
Low	1.24	1.14, 1.34	<0.001	<0.001
Hematocrit tertiles (admission)				
High	1.09	1.00, 1.19	0.060	0.107
Low	1.11	1.02, 1.20	0.017	0.054
Hematocrit tertiles (discharge)				
High	0.98	0.89, 1.07	0.608	0.695
Low	1.27	1.18, 1.38	<0.001	0.045
MCH tertiles (admission)				
High	0.99	0.91, 1.08	0.785	0.966
Low	1.09	1.01, 1.18	0.034	0.072
MCH tertiles (discharge)				
High	0.95	0.87, 1.03	0.227	0.330
Low	1.04	0.96, 1.13	0.323	0.431
MCHC tertiles (admission)				
High	0.98	0.90, 1.08	0.725	0.966
Low	1.17	1.08, 1.27	<0.001	<0.001
MCHC tertiles (discharge)				
High	0.92	0.84, 1.01	0.067	0.134
Low	1.14	1.05, 1.23	0.001	0.003
MCV tertiles (admission)				
High	1.00	0.92, 1.09	0.986	0.986
Low	1.00	0.92, 1.08	0.971	0.986
MCV tertiles (discharge)				
High	1.04	0.96, 1.13	0.359	0.442
Low	1.02	0.94, 1.10	0.709	0.755
Platelet count tertiles (admission)				
High	1.11	1.02, 1.21	0.012	0.048
Low	1.00	0.92, 1.09	0.920	0.986
Platelet count tertiles (discharge)				
High	1.15	1.06, 1.24	0.001	0.003
Low	1.06	0.98, 1.16	0.145	0.232
MPV tertiles (admission)				
High	1.09	1.01, 1.18	0.036	0.072
Low	1.03	0.95, 1.12	0.449	0.653
MPV tertiles (discharge)				
High	1.19	1.10, 1.29	<0.001	<0.001
Low	1.07	0.98, 1.17	0.108	0.192

The middle tertile served as the reference category for all hematological parameters and is therefore not displayed. Adjusted *p*-values were calculated using the Benjamini–Hochberg false-discovery-rate procedure. Abbreviations: CI, confidence interval; FDR, false discovery rate; HR, hazard ratio; MCH, mean corpuscular hemoglobin; MCHC, mean corpuscular hemoglobin concentration; MCV, mean corpuscular volume; MPV, mean platelet volume.

## Data Availability

Data will be shared on request to the corresponding author with permission of the Institute for Medical informatics, Statistics and Documentation of the Medical University of Graz.

## Supplementary Materials

The following supporting information can be downloaded at https://www.mdpi.com/article/10.3390/jcm15124455/s1. Table S1: Hemogram values overall, by tertiles and outcome; Table S2: Overall outcomes and follow-up time; Table S3: Missing data summary; Table S4: Multivariable Cox regression analysis of MACE with hematological marker tertiles at admission and discharge (complete case analysis); Figure S1: Kaplan–Meier Curves for 3-point MACE Stratified by Discharge Tertiles; Figure S2: Kaplan–Meier estimates of event probability for cardiovascular death, recurrent myocardial infarction, and stroke; Figure S3: Adjusted hazard ratios of MACE for hemogram values at discharge; Figure S4: Adjusted hazard ratios for recurrent myocardial infarction and stroke according to hematological marker tertiles at admission and discharge; Figure S5: Time-dependent receiver operating characteristic curves for the clinical base model plus the respective admission hematological marker shown in each panel.
