# Supplementary material for: Erythrocyte and Platelet Indices at Admission and Discharge Stratify Long-Term Cardiovascular Risk After Invasive Treatment of Myocardial Infarction
Source: J Clin Med. 2026 Jun 9;15(12):4455. doi: 10.3390/jcm15124455 (PMC13301232; doi:10.3390/jcm15124455)
Supplement: Supplementary file 1 [file jcm-15-04455-s001.zip › jcm-4310640-supplementary.pdf]

Supplementary Material

Table S1. Hemogram values overall, by tertiles and outcome

Characteristic	Overall N = 10,920[1]	1st Tertile	2nd Tertile	3rd Tertile	No MACE N = 7,480[1]	MACE N = 3,440[1]	p-value[2]
**Hemoglobin (admission) [g/dL]**	14.2 (13.0, 15.3)	12.4 (11.5, 13.0)	14.2 (13.9, 14.6)	15.8 (15.3, 16.4)	14.3 (13.2, 15.4)	13.8 (12.5, 15.0)	<0.001
**Hemoglobin (discharge) [g/dL]**	13.1 (11.7, 14.3)	11.0 (10.2, 11.7)	13.1 (12.7, 13.5)	14.8 (14.3, 15.4)	13.3 (12.0, 14.4)	12.6 (11.1, 13.9)	<0.001
**Erythrocytes (admission) [10^{12}/L]**	4.6 (4.3, 5.0)	4.1 (3.9, 4.3)	4.6 (4.5, 4.7)	5.1 (5.0, 5.3)	4.7 (4.3, 5.0)	4.6 (4.2, 4.9)	<0.001
**Erythrocytes (discharge) [10^{12}/L]**	4.3 (3.9, 4.7)	3.7 (3.4, 3.9)	4.3 (4.2, 4.5)	4.9 (4.7, 5.1)	4.4 (4.0, 4.7)	4.2 (3.7, 4.6)	<0.001
**Hematocrit (admission) [%]**	41.5 (38.3, 44.2)	37.0 (34.2, 38.3)	41.5 (40.6, 42.4)	45.7 (44.3, 47.3)	41.9 (38.9, 44.5)	40.8 (37.0, 44.0)	<0.001
**Hematocrit (discharge) [%]**	38.8 (35.0, 42.0)	33.3 (30.9, 35.0)	39.0 (37.8, 40.0)	43.6 (42.1, 45.1)	39.1 (35.9, 42.2)	37.5 (33.6, 41.0)	<0.001
**MCH (admission) [pg]**	30.5 (29.3, 31.7)	28.9 (28.0, 29.4)	30.5 (30.1, 31.0)	32.0 (31.7, 33.0)	30.6 (29.5, 31.7)	30.3 (29.0, 31.5)	<0.001
**MCH (discharge) [pg]**	30.3 (29.2, 31.4)	28.8 (28.0, 29.2)	30.4 (30.0, 30.8)	32.0 (31.6, 32.9)	30.4 (29.3, 31.5)	30.0 (29.0, 31.2)	<0.001
**MCHC (admission) [g/dL]**	34.0 (33.3, 35.0)	33.0 (32.3, 33.3)	34.1 (34.0, 34.4)	35.3 (35.0, 35.9)	34.1 (33.4, 35.0)	34.0 (33.0, 34.8)	<0.001
**MCHC (discharge) [g/dL]**	33.8 (33.0, 34.6)	32.8 (32.2, 33.0)	33.9 (33.6, 34.1)	35.0 (34.7, 35.4)	33.90 (33.1, 34.7)	33.6 (32.8, 34.3)	<0.001
**MCV (admission) [fL]**	89.00 (86.2, 92.1)	85.0 (83.0, 86.2)	89.0 (88.2, 90.0)	94.0 (92.2, 96.0)	89.0 (86.2, 92.0)	89.1 (86.2, 92.2)	0.618
**MCV (discharge) [fL]**	89.8 (86.8, 92.7)	85.5 (83.6, 86.9)	89.8 (88.9, 90.6)	94.0 (92.7, 96.0)	89.7 (86.9, 92.6)	89.9 (86.8, 92.9)	0.407
**Platelet count (admission) [10^9/L]**	228.0 (190.0, 273.0)	175.0 (154.0, 190.0)	228.0 (216.0, 242.0)	298.0 (274.0, 334.7)	228.0 (191.0, 272.0)	227.0 (188.0, 276.0)	0.618
**Platelet count (discharge) [10^9/L]**	234.0 (190.00, 296.00)	174.0 (151.0, 190.0)	234.0 (219.0, 251.0)	333.0 (297.0, 397.0)	233.0 (191.00, 292.00)	237.0 (189.00, 306.00)	0.148
**MPV (admission) [fL]**	10.0 (9.0, 10.8)	8.4 (7.5, 9.0)	10.0 (9.8, 10.3)	11.1 (10.8, 11.7)	10.0 (9.0, 10.7)	10.1 (9.0, 10.9)	<0.001
**MPV (discharge) [fL]**	10.1 (9.1, 11.0)	8.5 (7.7, 9.1)	10.1 (9.9, 10.4)	11.3 (11.0, 11.9)	10.1 (9.1, 10.9)	10.2 (9.2, 11.1)	<0.001

[1] Median (Q1, Q3); n (%)
[2] Wilcoxon rank sum test; Pearson's Chi-squared test
Abbreviations: MACE, major adverse cardiovascular event; MCH, mean corpuscular hemoglobin; MCHC, mean corpuscular hemoglobin concentration; MCV, mean corpuscular volume; MPV, mean platelet volume.

Table S2. Overall outcomes and follow-up time

Time	Cumulative incidence (%)
Follow-up time, months	103 (69, 138)
**MACE**	
Overall	31.5
3 months	8.4
6 months	10.1
12 months	12.3
5 years	24.0
10 years	32.0
**CV death**	
Overall	18.6
3 months	5.7
6 months	6.4
12 months	7.3
5 years	13.5
10 years	19.1
**Myocardial infarction**	
Overall	11.5
3 months	2.4
6 months	3.2
12 months	4.3
5 years	9.0
10 years	11.7
**Stroke**	
Overall	7.0
3 months	0.6
6 months	0.9
12 months	1.4
5 years	4.6
10 years	6.9
**All-cause death**	
Overall	36.5
3 months	7.5
6 months	8.8
12 months	10.5
5 years	23.0
10 years	36.4

Table S3. Missing data summary

Variable	N	n missing	% missing
Age	10920	0	0.0
Sex	10920	0	0.0
BMI	10920	3160	28.9
Hyperlipidemia	10920	0	0.0
Previous MI	10920	0	0.0
Previous Stroke	10920	0	0.0
Diabetes	10920	0	0.0
PCI–multivessel	10920	0	0.0
Hemoglobin (admission)	10920	89	0.8
Hemoglobin (discharge)	10920	89	0.8
Erythrocytes (admission)	10920	113	1.0
Erythrocytes (discharge)	10920	113	1.0
Hematocrit (admission)	10920	94	0.9
Hematocrit (discharge)	10920	94	0.9
MCH (admission)	10920	95	0.9
MCH (discharge)	10920	95	0.9
MCHC (admission)	10920	163	1.5
MCHC (discharge)	10920	163	1.5
MCV (admission)	10920	95	0.9
MCV (discharge)	10920	95	0.9
Platelet count (admission)	10920	95	0.9
Platelet count (discharge)	10920	95	0.9
MPV (admission)	10920	234	2.1
MPV (discharge)	10920	234	2.1

Table S4. Multivariable Cox regression analysis of MACE with hematological marker tertiles at admission and discharge (complete case analysis)

Characteristic	HR	95% CI	p-value
**Hemoglobin tertiles (admission)**			
High	1.20	1.05, 1.36	0.006
Low	1.30	1.15, 1.47	<0.001
**Hemoglobin tertiles (discharge)**			
High	0.91	0.80, 1.04	0.169
Low	1.26	1.12, 1.41	<0.001
**Erythrocytes tertiles (admission)**			
High	1.09	0.96, 1.24	0.162
Low	1.15	1.02, 1.29	0.019
**Erythrocytes tertiles (discharge)**			
High	1.01	0.89, 1.15	0.832
Low	1.28	1.14, 1.43	<0.001
**Hematocrit tertiles (admission)**			
High	1.13	1.00, 1.28	0.054
Low	1.19	1.06, 1.34	0.004
**Hematocrit tertiles (discharge)**			
High	1.01	0.89, 1.15	0.879
Low	1.31	1.17, 1.47	<0.001
**MCH tertiles (admission)**			
High	0.98	0.87, 1.11	0.793
Low	1.10	0.98, 1.24	0.094
**MCH tertiles (discharge)**			
High	0.97	0.85, 1.09	0.590
Low	1.06	0.94, 1.18	0.346
**MCHC tertiles (admission)**			
High	0.98	0.86, 1.11	0.702
Low	1.12	1.00, 1.25	0.056
**MCHC tertiles (discharge)**			
High	0.93	0.82, 1.06	0.284
Low	1.13	1.01, 1.27	0.029
**MCV tertiles (admission)**			
High	1.01	0.90, 1.14	0.881
Low	1.08	0.96, 1.21	0.211
**MCV tertiles (discharge)**			
High	1.04	0.92, 1.17	0.533
Low	1.05	0.94, 1.19	0.385
**Platelet count tertiles (admission)**			
High	1.12	1.00, 1.26	0.055
Low	0.96	0.86, 1.09	0.546
**Platelet count tertiles (discharge)**			
High	1.19	1.06, 1.34	0.003
Low	1.03	0.91, 1.17	0.630
**MPV tertiles (admission)**			
High	1.15	1.02, 1.29	0.019
Low	1.04	0.92, 1.17	0.565
**MPV tertiles (discharge)**			
High	1.11	0.99, 1.25	0.074
Low	1.02	0.90, 1.14	0.792

The middle tertile served as the reference category for all hematological parameters and is therefore not displayed.
Abbreviations: CI, confidence interval; HR, hazard ratio; MCH, mean corpuscular hemoglobin; MCHC, mean corpuscular hemoglobin concentration; MCV, mean corpuscular volume; MPV, mean platelet volume

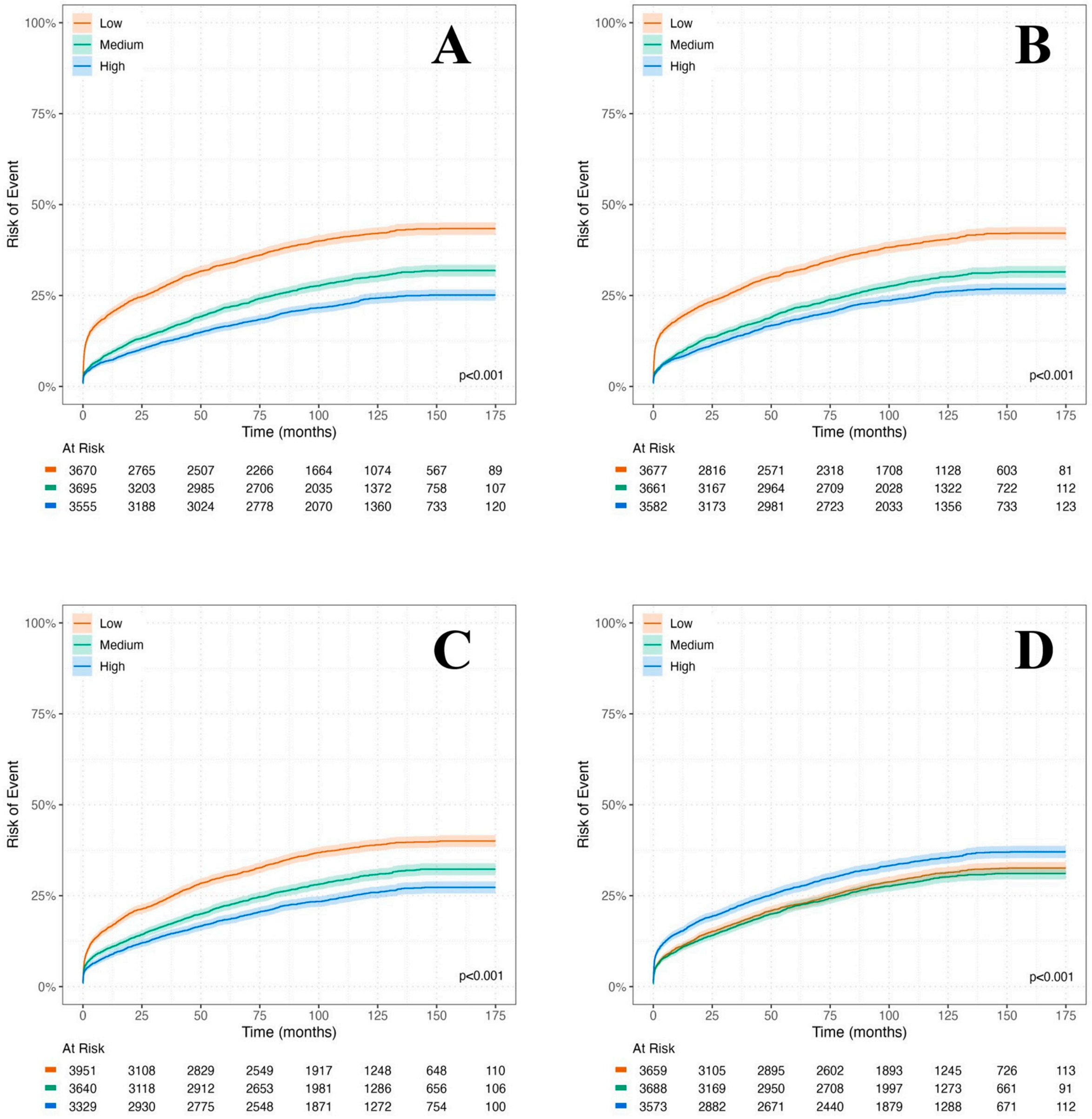

Figure S1. Kaplan-Meier Curves for 3-point MACE Stratified by Discharge Tertiles. (A) Hemoglobin, (B) erythrocyte count, (C) MCHC, and (D) MPV at discharge are shown in three groups (low, medium, high). Abbreviations: MACE, major adverse cardiovascular events; MCH, mean corpuscular hemoglobin; MCHC, mean corpuscular hemoglobin concentration; MCV, mean corpuscular volume; MPV, mean platelet volume.

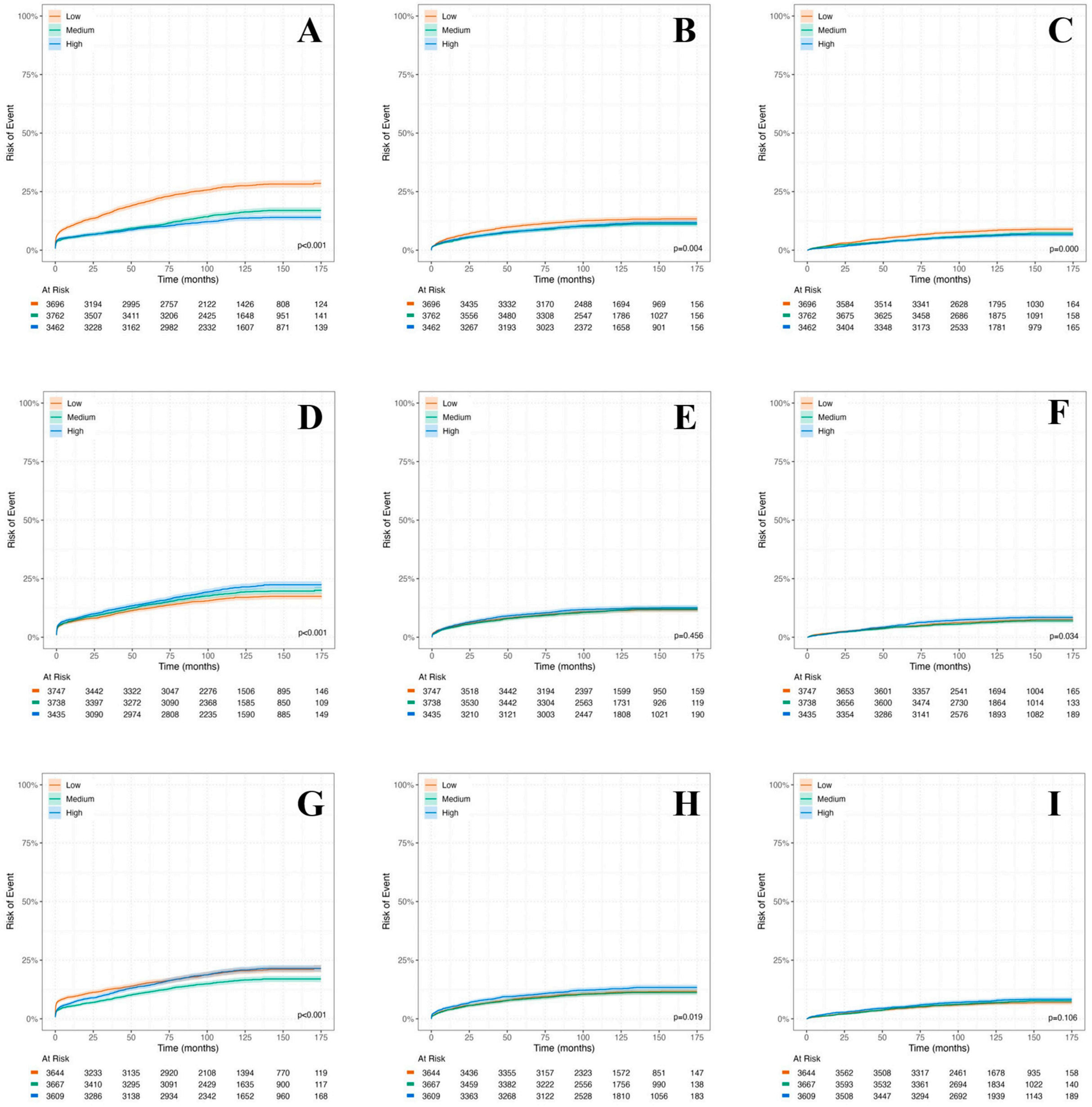

Figure S2. Kaplan-Meier estimates of event probability for cardiovascular death, recurrent myocardial infarction, and stroke. (A–C) CV death, recurrent MI, and stroke according to admission hemoglobin tertiles. (D–F) CV death, recurrent MI, and stroke according to admission MPV tertiles. (G–I) CV death, recurrent MI, and stroke according to discharge platelet count tertiles (low, medium, high).

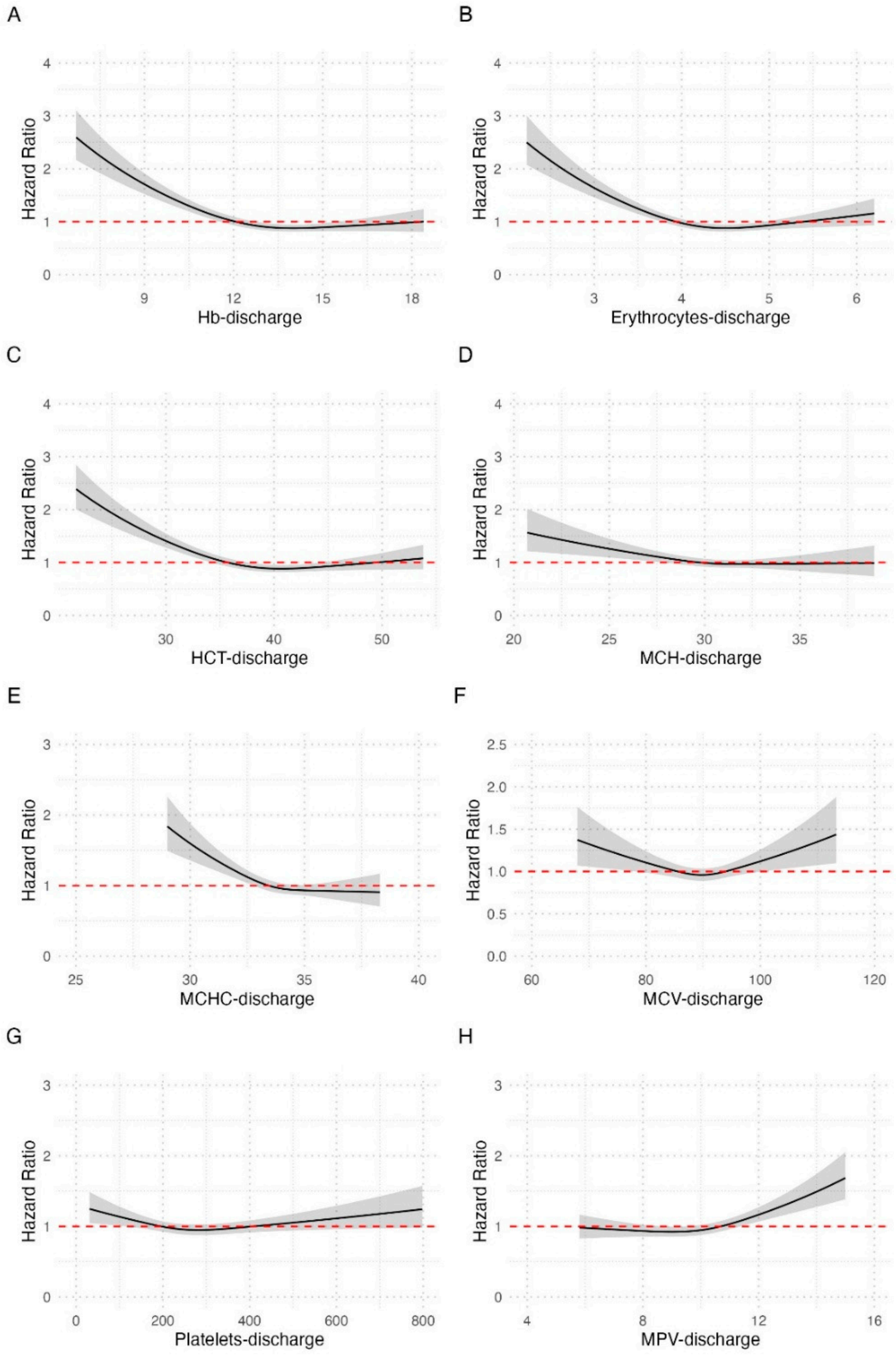

Figure S3. Adjusted hazard ratios of 3-point MACE for hemogram values at discharge. (A) hemoglobin, (B) erythrocyte count, (C) hematocrit, (D) MCH, (E) MCHC, (F) MCV, (G) platelet count, and (H) MPV. Abbreviations: Hb, hemoglobin; HR, hazard ratio; MACE, major adverse cardiovascular events; MCH, mean corpuscular hemoglobin; MCHC, mean corpuscular hemoglobin concentration; MCV, mean corpuscular volume; MPV, mean platelet volume.

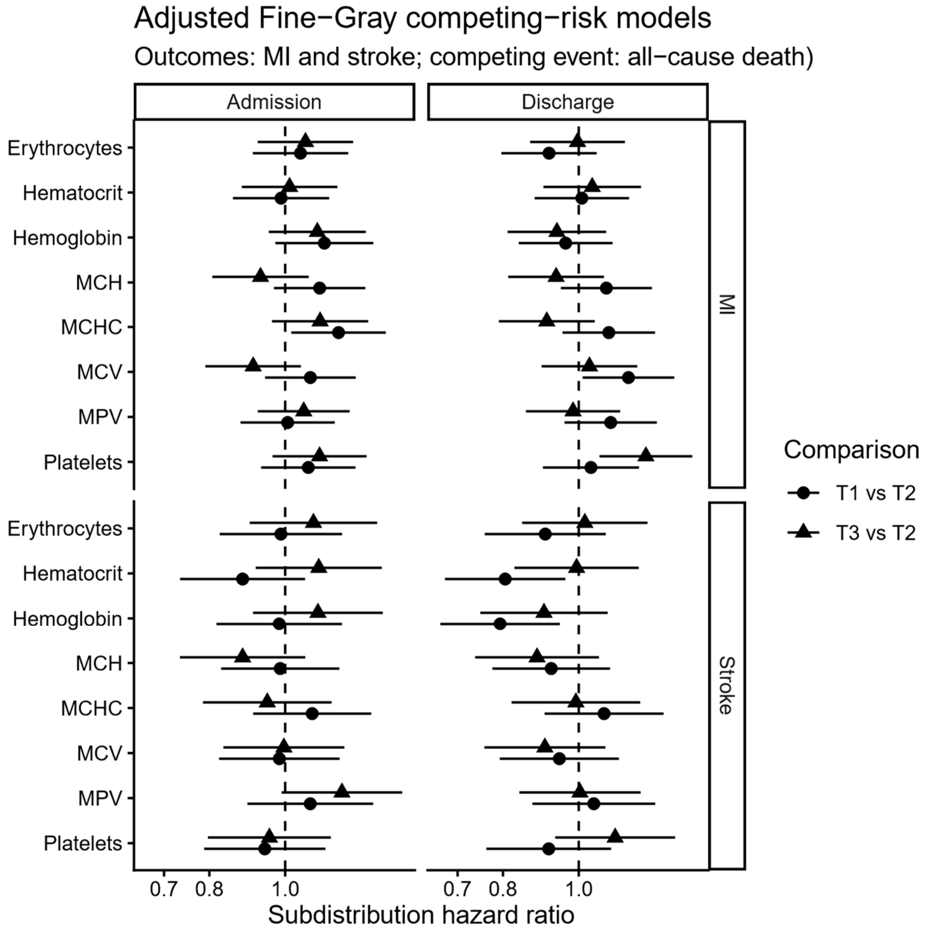

Figure S4. Adjusted hazard ratios for recurrent myocardial infarction and stroke according to hematological marker tertiles at admission and discharge. All-cause death before the respective non-fatal endpoint was treated as the competing event. T2 represents the middle tertile and served as the reference category; T1 represents the low tertile and T3 the high tertile. Models were adjusted for age, sex, BMI, hyperlipidemia, MI type, previous MI, previous stroke, diabetes, multiple- versus single-vessel PCI, cumulative comorbidity burden, and eGFR. Abbreviations: MI, myocardial infarction; MCH, mean corpuscular hemoglobin; MCHC, mean corpuscular hemoglobin concentration; MCV, mean corpuscular volume; MPV, mean platelet volume.

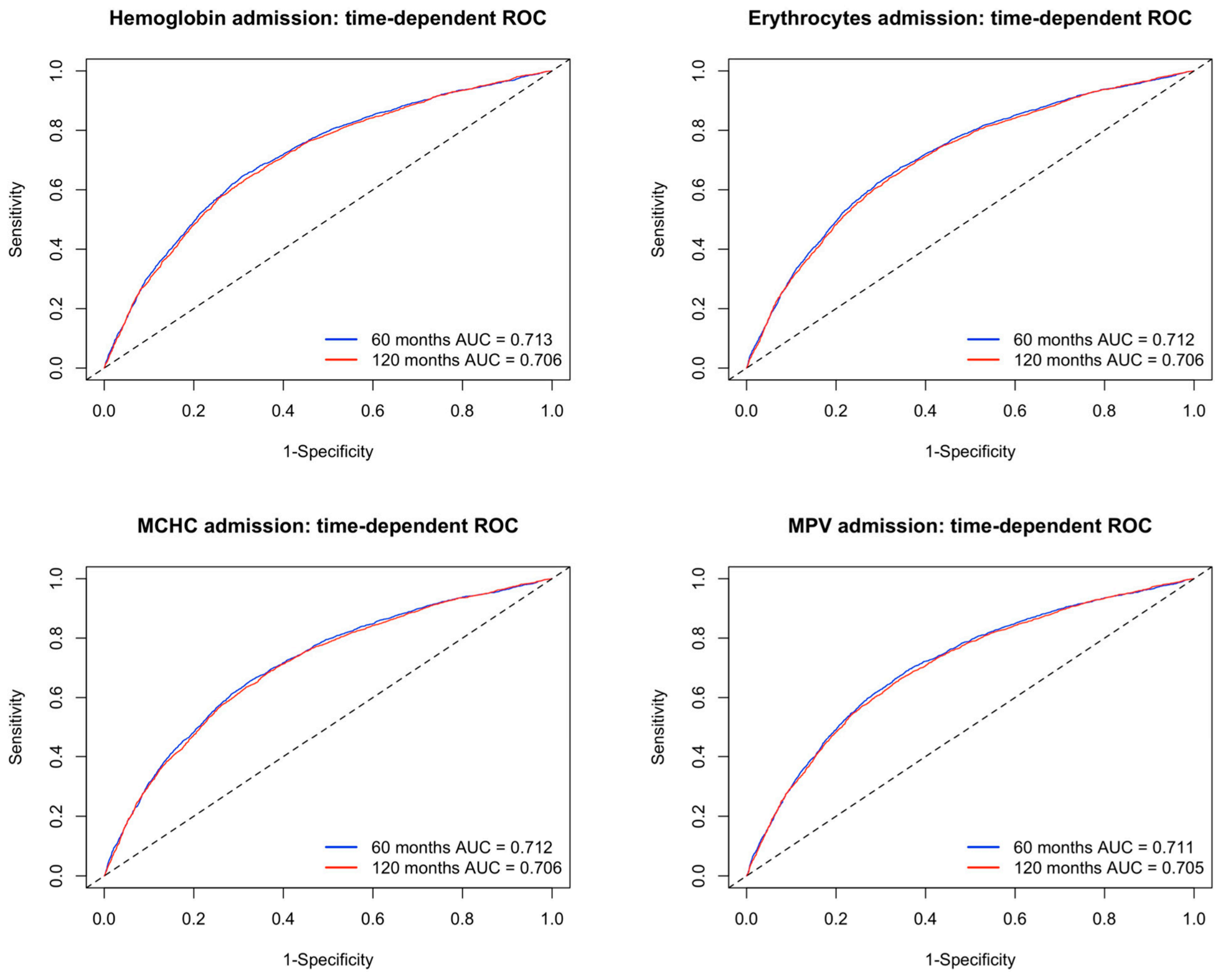

Figure S5. Time-dependent receiver operating characteristic curves for the clinical base model plus the respective admission hematological marker shown in each panel. Curves are displayed for prediction of 3-point MACE at 60 months and 120 months. The clinical base model included age, sex, BMI, hyperlipidemia, MI type, previous MI, previous stroke, diabetes, multiple- versus single-vessel PCI, number of baseline comorbidities, and eGFR. AUC values are shown within each panel. Abbreviations: AUC, area under the curve; MACE, major adverse cardiovascular events; MCHC, mean corpuscular hemoglobin concentration; MPV, mean platelet volume; ROC, receiver operating characteristic.
